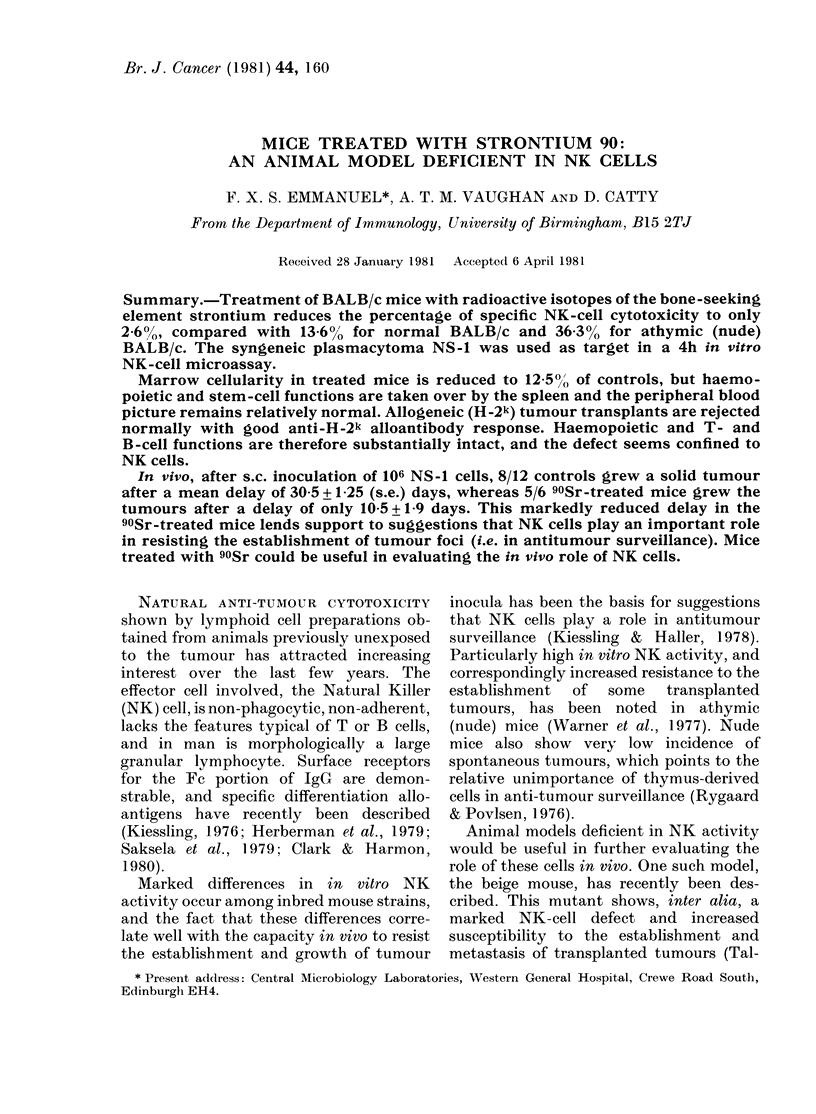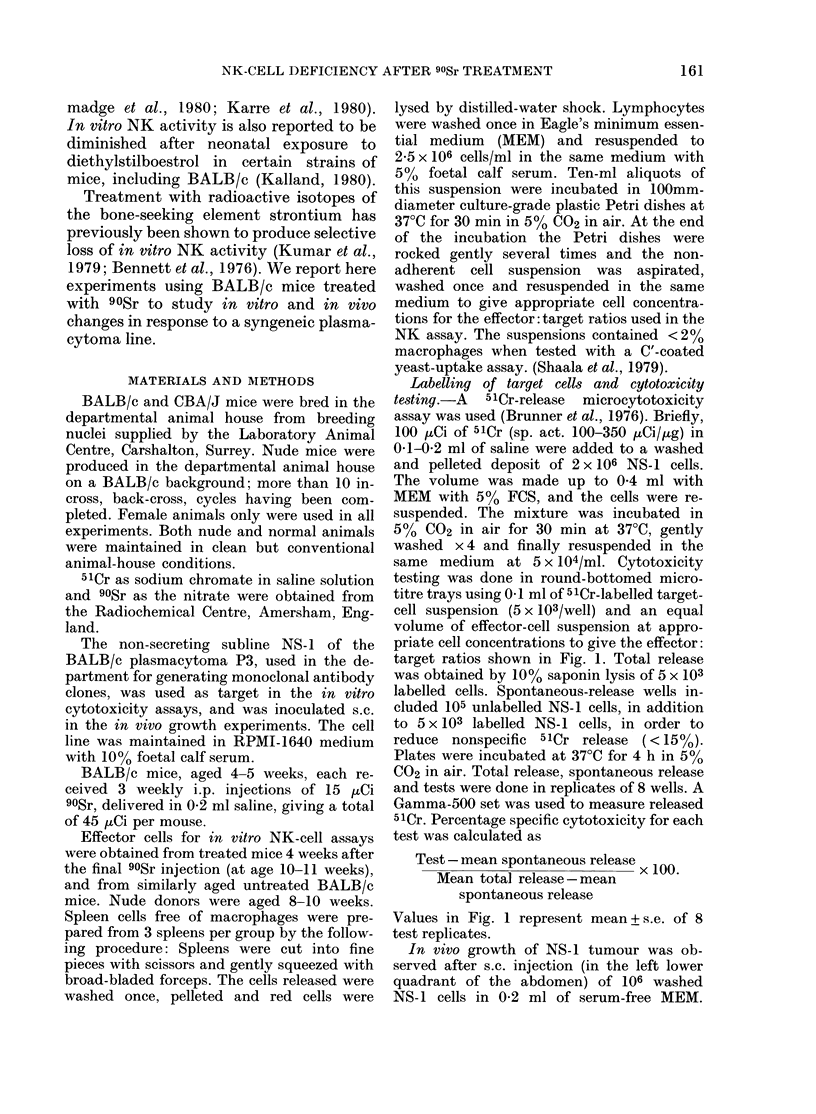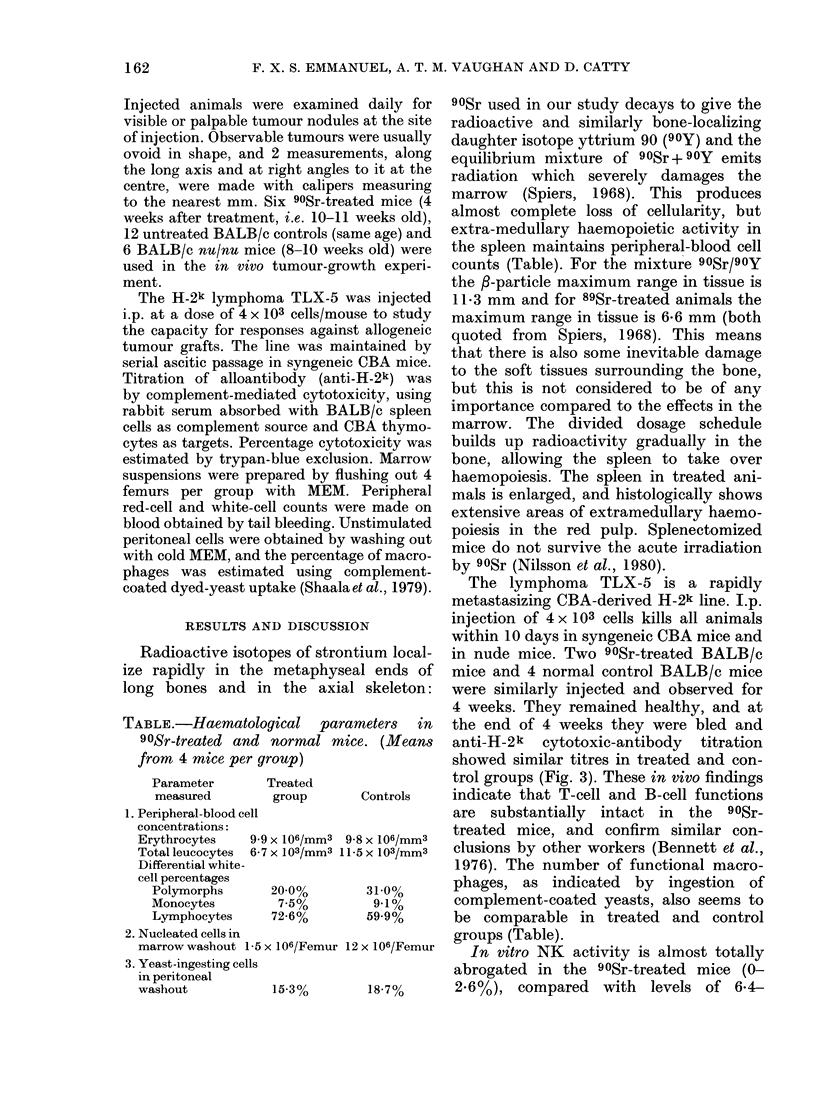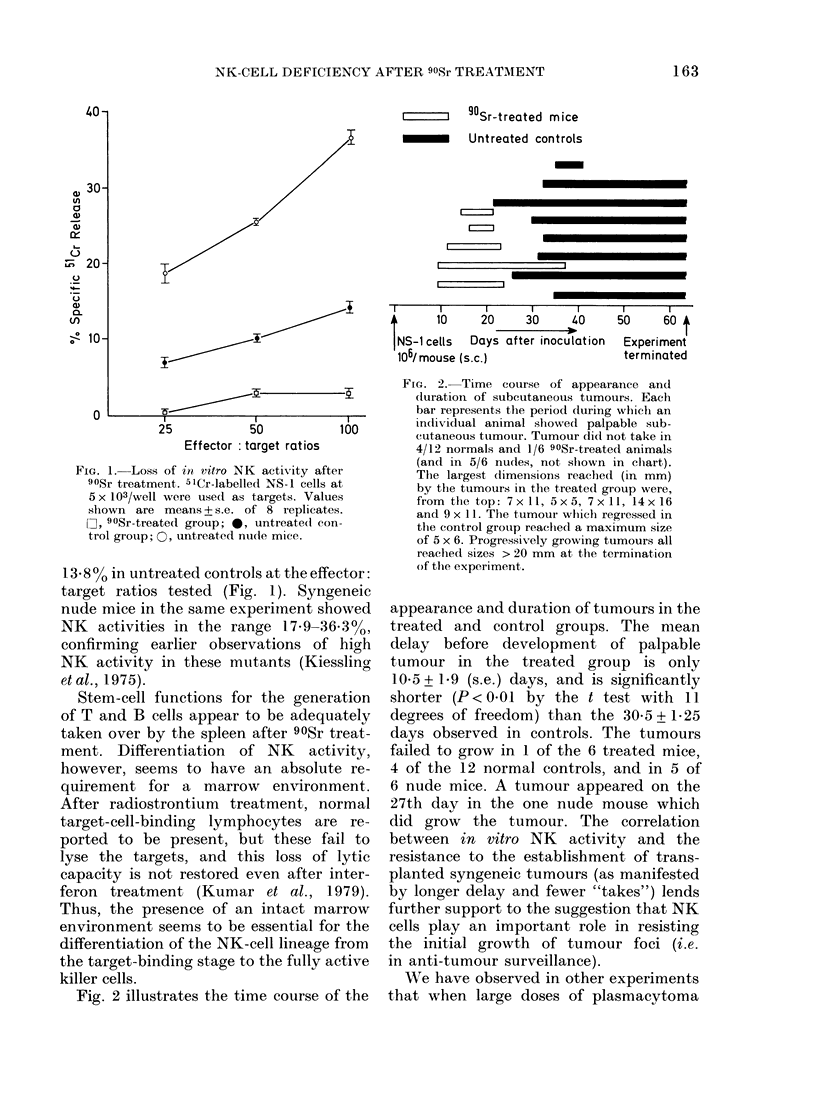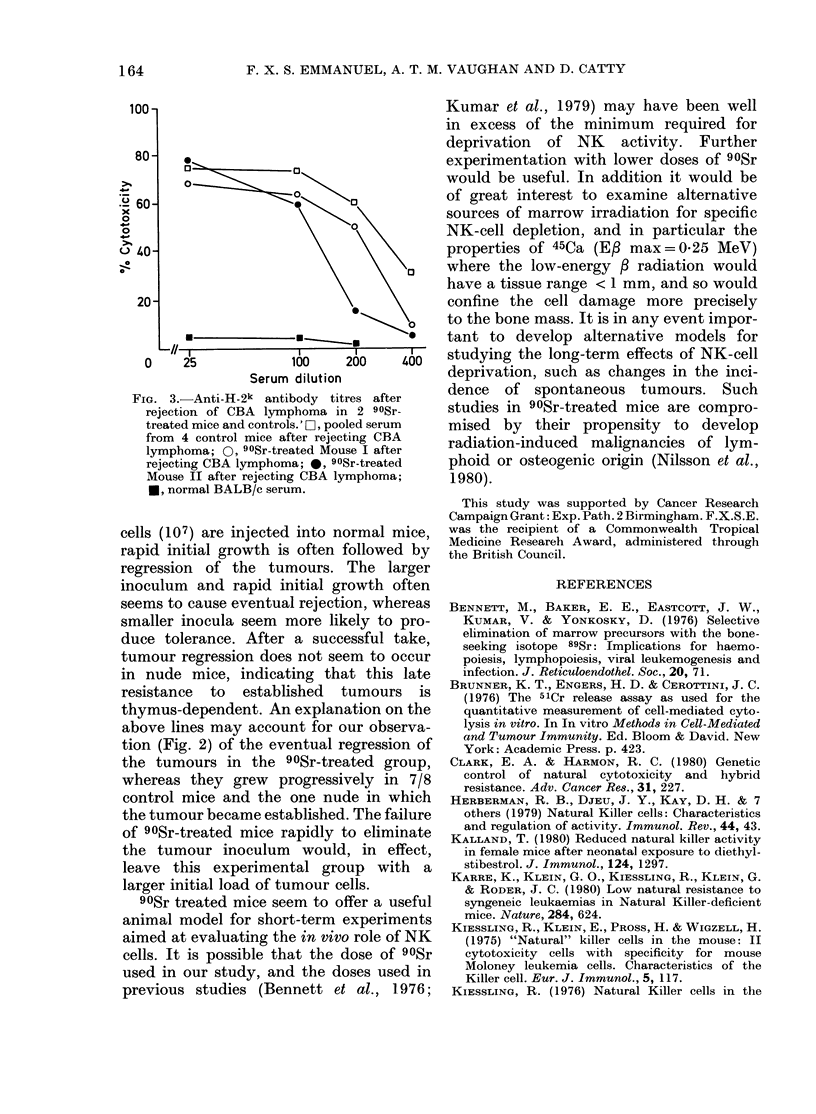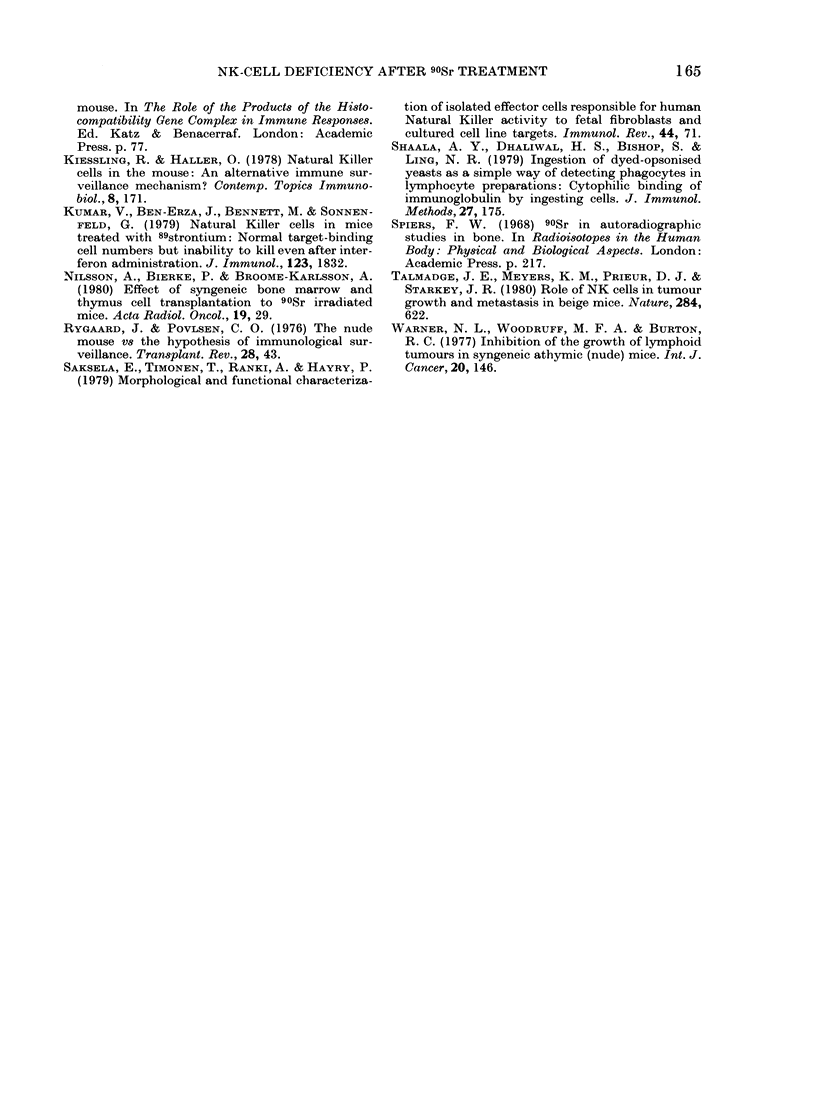# Mice treated with strontium 90: an animal model deficient in NK cells.

**DOI:** 10.1038/bjc.1981.166

**Published:** 1981-08

**Authors:** F. X. Emmanuel, A. T. Vaughan, D. Catty

## Abstract

Treatment of BALB/c mice with radioactive isotopes of the bone-seeking element strontium reduces the percentage of specific NK-cell cytotoxicity to only 2.6%, compared with 13.6% for normal BALB/c and 36.3% for athymic (nude) BALB/c. The syngeneic plasmacytoma NS-1 was used as target in a 4th in vitro NK-cell microassay. Marrow cellularity in treated mice is reduced to 12.5% of controls, but haemopoietic and stem-cell functions are taken over by the spleen and the peripheral blood picture remains relatively normal. Allogenic (H-2k) tumour transplants are rejected normally with good anti-H-2k alloantibody response. Haemopoietic and T- and B-cell functions are therefore substantially intact, and the defect seems confined to NK cells. In vivo, after s.c. inoculation of 10(6) NS-1 cells, 8/12 controls grew a solid tumour after a mean delay of 30.5 +/- 1.25 (s.e.) days, whereas 5/6 90Sr-treated mice grew the tumours after a delay of only 10.5 +/- 1.8 days. This markedly reduced delay in the 90Sr-treated mice lends support to suggestions that NK cells play an important role in resisting the establishment of tumour foci (i.e. in antitumour surveillance). Mice treated with 90Sr could be useful in evaluating the in vitro role of NK cells.


*Br. J. Cancer* (1981) **44**, 160

### MICE TREATED WITH STRONTIUM 90: AN ANIMAL MODEL DEFICIENT IN NK CELLS

F. X. S. EMMANUEL*, A. T. M. VAUGHAN AND D. CATTY

*From the Department of Immunology, University of Birmingham, B15 2TJ*

Received 28 January 1981 Accepted 6 April 1981

**Summary.—Treatment of BALB/c mice with radioactive isotopes of the bone-seeking element strontium reduces the percentage of specific NK-cell cytotoxicity to only 2·6%, compared with 13·6% for normal BALB/c and 36·3% for athymic (nude) BALB/c. The syngeneic plasmacytoma NS-1 was used as target in a 4h *in vitro* NK-cell microassay.**

**Marrow cellularity in treated mice is reduced to 12·5% of controls, but haemopoietic and stem-cell functions are taken over by the spleen and the peripheral blood picture remains relatively normal. Allogeneic (H-2$^k$) tumour transplants are rejected normally with good anti-H-2$^k$ alloantibody response. Haemopoietic and T- and B-cell functions are therefore substantially intact, and the defect seems confined to NK cells.**

***In vivo*, after s.c. inoculation of $10^6$ NS-1 cells, 8/12 controls grew a solid tumour after a mean delay of 30·5 ± 1·25 (s.e.) days, whereas 5/6 $^{90}$Sr-treated mice grew the tumours after a delay of only 10·5 ± 1·9 days. This markedly reduced delay in the $^{90}$Sr-treated mice lends support to suggestions that NK cells play an important role in resisting the establishment of tumour foci (*i.e.* in antitumour surveillance). Mice treated with $^{90}$Sr could be useful in evaluating the *in vivo* role of NK cells.**

NATURAL ANTI-TUMOUR CYTOTOXICITY shown by lymphoid cell preparations obtained from animals previously unexposed to the tumour has attracted increasing interest over the last few years. The effector cell involved, the Natural Killer (NK) cell, is non-phagocytic, non-adherent, lacks the features typical of T or B cells, and in man is morphologically a large granular lymphocyte. Surface receptors for the Fc portion of IgG are demonstrable, and specific differentiation alloantigens have recently been described (Kiessling, 1976; Herberman *et al.*, 1979; Saksela *et al.*, 1979; Clark & Harmon, 1980).

Marked differences in *in vitro* NK activity occur among inbred mouse strains, and the fact that these differences correlate well with the capacity *in vivo* to resist the establishment and growth of tumour inocula has been the basis for suggestions that NK cells play a role in antitumour surveillance (Kiessling & Haller, 1978). Particularly high *in vitro* NK activity, and correspondingly increased resistance to the establishment of some transplanted tumours, has been noted in athymic (nude) mice (Warner *et al.*, 1977). Nude mice also show very low incidence of spontaneous tumours, which points to the relative unimportance of thymus-derived cells in anti-tumour surveillance (Rygaard & Povlsen, 1976).

Animal models deficient in NK activity would be useful in further evaluating the role of these cells *in vivo*. One such model, the beige mouse, has recently been described. This mutant shows, *inter alia*, a marked NK-cell defect and increased susceptibility to the establishment and metastasis of transplanted tumours (Tal-

* Present address: Central Microbiology Laboratories, Western General Hospital, Crewe Road South, Edinburgh EH4.

madge *et al.*, 1980; Karre *et al.*, 1980). *In vitro* NK activity is also reported to be diminished after neonatal exposure to diethylstilboestrol in certain strains of mice, including BALB/c (Kalland, 1980).

Treatment with radioactive isotopes of the bone-seeking element strontium has previously been shown to produce selective loss of *in vitro* NK activity (Kumar *et al.*, 1979; Bennett *et al.*, 1976). We report here experiments using BALB/c mice treated with $^{90}Sr$ to study *in vitro* and *in vivo* changes in response to a syngeneic plasmacytoma line.

### MATERIALS AND METHODS

BALB/c and CBA/J mice were bred in the departmental animal house from breeding nuclei supplied by the Laboratory Animal Centre, Carshalton, Surrey. Nude mice were produced in the departmental animal house on a BALB/c background; more than 10 incross, back-cross, cycles having been completed. Female animals only were used in all experiments. Both nude and normal animals were maintained in clean but conventional animal-house conditions.

$^{51}Cr$ as sodium chromate in saline solution and $^{90}Sr$ as the nitrate were obtained from the Radiochemical Centre, Amersham, England.

The non-secreting subline NS-1 of the BALB/c plasmacytoma P3, used in the department for generating monoclonal antibody clones, was used as target in the *in vitro* cytotoxicity assays, and was inoculated s.c. in the *in vivo* growth experiments. The cell line was maintained in RPMI-1640 medium with 10% foetal calf serum.

BALB/c mice, aged 4–5 weeks, each received 3 weekly i.p. injections of 15 $\mu$Ci $^{90}Sr$, delivered in 0·2 ml saline, giving a total of 45 $\mu$Ci per mouse.

Effector cells for *in vitro* NK-cell assays were obtained from treated mice 4 weeks after the final $^{90}Sr$ injection (at age 10–11 weeks), and from similarly aged untreated BALB/c mice. Nude donors were aged 8–10 weeks. Spleen cells free of macrophages were prepared from 3 spleens per group by the following procedure: Spleens were cut into fine pieces with scissors and gently squeezed with broad-bladed forceps. The cells released were washed once, pelleted and red cells were lysed by distilled-water shock. Lymphocytes were washed once in Eagle's minimum essential medium (MEM) and resuspended to $2{\cdot}5 \times 10^6$ cells/ml in the same medium with 5% foetal calf serum. Ten-ml aliquots of this suspension were incubated in 100mm-diameter culture-grade plastic Petri dishes at 37°C for 30 min in 5% $CO_2$ in air. At the end of the incubation the Petri dishes were rocked gently several times and the non-adherent cell suspension was aspirated, washed once and resuspended in the same medium to give appropriate cell concentrations for the effector:target ratios used in the NK assay. The suspensions contained $<2\%$ macrophages when tested with a C′-coated yeast-uptake assay. (Shaala *et al.*, 1979).

*Labelling of target cells and cytotoxicity testing.*—A $^{51}Cr$-release microcytotoxicity assay was used (Brunner *et al.*, 1976). Briefly, 100 $\mu$Ci of $^{51}Cr$ (sp. act. 100–350 $\mu$Ci/$\mu$g) in 0·1–0·2 ml of saline were added to a washed and pelleted deposit of $2 \times 10^6$ NS-1 cells. The volume was made up to 0·4 ml with MEM with 5% FCS, and the cells were resuspended. The mixture was incubated in 5% $CO_2$ in air for 30 min at 37°C, gently washed $\times 4$ and finally resuspended in the same medium at $5 \times 10^4$/ml. Cytotoxicity testing was done in round-bottomed microtitre trays using 0·1 ml of $^{51}Cr$-labelled target-cell suspension ($5 \times 10^3$/well) and an equal volume of effector-cell suspension at appropriate cell concentrations to give the effector: target ratios shown in Fig. 1. Total release was obtained by 10% saponin lysis of $5 \times 10^3$ labelled cells. Spontaneous-release wells included $10^5$ unlabelled NS-1 cells, in addition to $5 \times 10^3$ labelled NS-1 cells, in order to reduce nonspecific $^{51}Cr$ release ($<15\%$). Plates were incubated at 37°C for 4 h in 5% $CO_2$ in air. Total release, spontaneous release and tests were done in replicates of 8 wells. A Gamma-500 set was used to measure released $^{51}Cr$. Percentage specific cytotoxicity for each test was calculated as

$$\frac{\text{Test} - \text{mean spontaneous release}}{\text{Mean total release} - \text{mean spontaneous release}} \times 100.$$

Values in Fig. 1 represent mean $\pm$ s.e. of 8 test replicates.

*In vivo* growth of NS-1 tumour was observed after s.c. injection (in the left lower quadrant of the abdomen) of $10^6$ washed NS-1 cells in 0·2 ml of serum-free MEM.

Injected animals were examined daily for visible or palpable tumour nodules at the site of injection. Observable tumours were usually ovoid in shape, and 2 measurements, along the long axis and at right angles to it at the centre, were made with calipers measuring to the nearest mm. Six $^{90}$Sr-treated mice (4 weeks after treatment, *i.e.* 10–11 weeks old), 12 untreated BALB/c controls (same age) and 6 BALB/c *nu/nu* mice (8–10 weeks old) were used in the *in vivo* tumour-growth experiment.

The H-2$^{k}$ lymphoma TLX-5 was injected i.p. at a dose of $4 \times 10^3$ cells/mouse to study the capacity for responses against allogeneic tumour grafts. The line was maintained by serial ascitic passage in syngeneic CBA mice. Titration of alloantibody (anti-H-2$^{k}$) was by complement-mediated cytotoxicity, using rabbit serum absorbed with BALB/c spleen cells as complement source and CBA thymocytes as targets. Percentage cytotoxicity was estimated by trypan-blue exclusion. Marrow suspensions were prepared by flushing out 4 femurs per group with MEM. Peripheral red-cell and white-cell counts were made on blood obtained by tail bleeding. Unstimulated peritoneal cells were obtained by washing out with cold MEM, and the percentage of macrophages was estimated using complement-coated dyed-yeast uptake (Shaala *et al.*, 1979).

### RESULTS AND DISCUSSION

Radioactive isotopes of strontium localize rapidly in the metaphyseal ends of long bones and in the axial skeleton: $^{90}$Sr used in our study decays to give the radioactive and similarly bone-localizing daughter isotope yttrium 90 ($^{90}$Y) and the equilibrium mixture of $^{90}$Sr + $^{90}$Y emits radiation which severely damages the marrow (Spiers, 1968). This produces almost complete loss of cellularity, but extra-medullary haemopoietic activity in the spleen maintains peripheral-blood cell counts (Table). For the mixture $^{90}$Sr/$^{90}$Y the $\beta$-particle maximum range in tissue is 11·3 mm and for $^{89}$Sr-treated animals the maximum range in tissue is 6·6 mm (both quoted from Spiers, 1968). This means that there is also some inevitable damage to the soft tissues surrounding the bone, but this is not considered to be of any importance compared to the effects in the marrow. The divided dosage schedule builds up radioactivity gradually in the bone, allowing the spleen to take over haemopoiesis. The spleen in treated animals is enlarged, and histologically shows extensive areas of extramedullary haemopoiesis in the red pulp. Splenectomized mice do not survive the acute irradiation by $^{90}$Sr (Nilsson *et al.*, 1980).

TABLE.—*Haematological parameters in $^{90}$Sr-treated and normal mice. (Means from 4 mice per group)*

| Parameter measured | Treated group | Controls |
|---|---|---|
| 1. Peripheral-blood cell concentrations: | | |
| Erythrocytes | $9·9 \times 10^6$/mm$^3$ | $9·8 \times 10^6$/mm$^3$ |
| Total leucocytes | $6·7 \times 10^3$/mm$^3$ | $11·5 \times 10^3$/mm$^3$ |
| Differential white-cell percentages | | |
| Polymorphs | 20·0% | 31·0% |
| Monocytes | 7·5% | 9·1% |
| Lymphocytes | 72·6% | 59·9% |
| 2. Nucleated cells in marrow washout | $1·5 \times 10^6$/Femur | $12 \times 10^6$/Femur |
| 3. Yeast-ingesting cells in peritoneal washout | 15·3% | 18·7% |

The lymphoma TLX-5 is a rapidly metastasizing CBA-derived H-2$^{k}$ line. I.p. injection of $4 \times 10^3$ cells kills all animals within 10 days in syngeneic CBA mice and in nude mice. Two $^{90}$Sr-treated BALB/c mice and 4 normal control BALB/c mice were similarly injected and observed for 4 weeks. They remained healthy, and at the end of 4 weeks they were bled and anti-H-2$^{k}$ cytotoxic-antibody titration showed similar titres in treated and control groups (Fig. 3). These *in vivo* findings indicate that T-cell and B-cell functions are substantially intact in the $^{90}$Sr-treated mice, and confirm similar conclusions by other workers (Bennett *et al.*, 1976). The number of functional macrophages, as indicated by ingestion of complement-coated yeasts, also seems to be comparable in treated and control groups (Table).

*In vitro* NK activity is almost totally abrogated in the $^{90}$Sr-treated mice (0–2·6%), compared with levels of 6·4–

FIG. 1.—Loss of *in vitro* NK activity after $^{90}$Sr treatment. $^{51}$Cr-labelled NS-1 cells at $5 \times 10^3$/well were used as targets. Values shown are means ± s.e. of 8 replicates. □, $^{90}$Sr-treated group; ●, untreated control group; ○, untreated nude mice.

FIG. 2.—Time course of appearance and duration of subcutaneous tumours. Each bar represents the period during which an individual animal showed palpable subcutaneous tumour. Tumour did not take in 4/12 normals and 1/6 $^{90}$Sr-treated animals (and in 5/6 nudes, not shown in chart). The largest dimensions reached (in mm) by the tumours in the treated group were, from the top: $7 \times 11$, $5 \times 5$, $7 \times 11$, $14 \times 16$ and $9 \times 11$. The tumour which regressed in the control group reached a maximum size of $5 \times 6$. Progressively growing tumours all reached sizes $> 20$ mm at the termination of the experiment.

13·8% in untreated controls at the effector: target ratios tested (Fig. 1). Syngeneic nude mice in the same experiment showed NK activities in the range 17·9–36·3%, confirming earlier observations of high NK activity in these mutants (Kiessling *et al.*, 1975).

Stem-cell functions for the generation of T and B cells appear to be adequately taken over by the spleen after $^{90}$Sr treatment. Differentiation of NK activity, however, seems to have an absolute requirement for a marrow environment. After radiostrontium treatment, normal target-cell-binding lymphocytes are reported to be present, but these fail to lyse the targets, and this loss of lytic capacity is not restored even after interferon treatment (Kumar *et al.*, 1979). Thus, the presence of an intact marrow environment seems to be essential for the differentiation of the NK-cell lineage from the target-binding stage to the fully active killer cells.

Fig. 2 illustrates the time course of the appearance and duration of tumours in the treated and control groups. The mean delay before development of palpable tumour in the treated group is only 10·5 ± 1·9 (s.e.) days, and is significantly shorter ($P < 0{\cdot}01$ by the $t$ test with 11 degrees of freedom) than the 30·5 ± 1·25 days observed in controls. The tumours failed to grow in 1 of the 6 treated mice, 4 of the 12 normal controls, and in 5 of 6 nude mice. A tumour appeared on the 27th day in the one nude mouse which did grow the tumour. The correlation between *in vitro* NK activity and the resistance to the establishment of transplanted syngeneic tumours (as manifested by longer delay and fewer "takes") lends further support to the suggestion that NK cells play an important role in resisting the initial growth of tumour foci (*i.e.* in anti-tumour surveillance).

We have observed in other experiments that when large doses of plasmacytoma

FIG. 3.—Anti-H-$2^k$ antibody titres after rejection of CBA lymphoma in 2 $^{90}$Sr-treated mice and controls.' □, pooled serum from 4 control mice after rejecting CBA lymphoma; ○, $^{90}$Sr-treated Mouse I after rejecting CBA lymphoma; ●, $^{90}$Sr-treated Mouse II after rejecting CBA lymphoma; ■, normal BALB/c serum.

cells ($10^7$) are injected into normal mice, rapid initial growth is often followed by regression of the tumours. The larger inoculum and rapid initial growth often seems to cause eventual rejection, whereas smaller inocula seem more likely to produce tolerance. After a successful take, tumour regression does not seem to occur in nude mice, indicating that this late resistance to established tumours is thymus-dependent. An explanation on the above lines may account for our observation (Fig. 2) of the eventual regression of the tumours in the $^{90}$Sr-treated group, whereas they grew progressively in 7/8 control mice and the one nude in which the tumour became established. The failure of $^{90}$Sr-treated mice rapidly to eliminate the tumour inoculum would, in effect, leave this experimental group with a larger initial load of tumour cells.

$^{90}$Sr treated mice seem to offer a useful animal model for short-term experiments aimed at evaluating the *in vivo* role of NK cells. It is possible that the dose of $^{90}$Sr used in our study, and the doses used in previous studies (Bennett *et al.*, 1976; Kumar *et al.*, 1979) may have been well in excess of the minimum required for deprivation of NK activity. Further experimentation with lower doses of $^{90}$Sr would be useful. In addition it would be of great interest to examine alternative sources of marrow irradiation for specific NK-cell depletion, and in particular the properties of $^{45}$Ca ($E\beta$ max = 0·25 MeV) where the low-energy $\beta$ radiation would have a tissue range < 1 mm, and so would confine the cell damage more precisely to the bone mass. It is in any event important to develop alternative models for studying the long-term effects of NK-cell deprivation, such as changes in the incidence of spontaneous tumours. Such studies in $^{90}$Sr-treated mice are compromised by their propensity to develop radiation-induced malignancies of lymphoid or osteogenic origin (Nilsson *et al.*, 1980).

This study was supported by Cancer Research Campaign Grant: Exp. Path. 2 Birmingham. F.X.S.E. was the recipient of a Commonwealth Tropical Medicine Researeh Award, administered through the British Council.

### REFERENCES

BENNETT, M., BAKER, E. E., EASTCOTT, J. W., KUMAR, V. & YONKOSKY, D. (1976) Selective elimination of marrow precursors with the bone-seeking isotope $^{89}$Sr: Implications for haemopoiesis, lymphopoiesis, viral leukemogenesis and infection. *J. Reticuloendothel. Soc.*, **20**, 71.

BRUNNER, K. T., ENGERS, H. D. & CEROTTINI, J. C. (1976) The $^{51}$Cr release assay as used for the quantitative measurement of cell-mediated cytolysis *in vitro*. In In vitro *Methods in Cell-Mediated and Tumour Immunity*. Ed. Bloom & David. New York: Academic Press. p. 423.

CLARK, E. A. & HARMON, R. C. (1980) Genetic control of natural cytotoxicity and hybrid resistance. *Adv. Cancer Res.*, **31**, 227.

HERBERMAN, R. B., DJEU, J. Y., KAY, D. H. & 7 others (1979) Natural Killer cells: Characteristics and regulation of activity. *Immunol. Rev.*, **44**, 43.

KALLAND, T. (1980) Reduced natural killer activity in female mice after neonatal exposure to diethylstibestrol. *J. Immunol.*, **124**, 1297.

KARRE, K., KLEIN, G. O., KIESSLING, R., KLEIN, G. & RODER, J. C. (1980) Low natural resistance to syngeneic leukaemias in Natural Killer-deficient mice. *Nature*, **284**, 624.

KIESSLING, R., KLEIN, E., PROSS, H. & WIGZELL, H. (1975) "Natural" killer cells in the mouse: II cytotoxicity cells with specificity for mouse Moloney leukemia cells. Characteristics of the Killer cell. *Eur. J. Immunol.*, **5**, 117.

KIESSLING, R. (1976) Natural Killer cells in the mouse. In *The Role of the Products of the Histocompatibility Gene Complex in Immune Responses.* Ed. Katz & Benacerraf. London: Academic Press. p. 77.

KIESSLING, R. & HALLER, O. (1978) Natural Killer cells in the mouse: An alternative immune surveillance mechanism? *Contemp. Topics Immunobiol.*, **8**, 171.

KUMAR, V., BEN-ERZA, J., BENNETT, M. & SONNENFELD, G. (1979) Natural Killer cells in mice treated with $^{89}$strontium: Normal target-binding cell numbers but inability to kill even after interferon administration. *J. Immunol.*, **123**, 1832.

NILSSON, A., BIERKE, P. & BROOME-KARLSSON, A. (1980) Effect of syngeneic bone marrow and thymus cell transplantation to $^{90}$Sr irradiated mice. *Acta Radiol. Oncol.*, **19**, 29.

RYGAARD, J. & POVLSEN, C. O. (1976) The nude mouse *vs* the hypothesis of immunological surveillance. *Transplant. Rev.*, **28**, 43.

SAKSELA, E., TIMONEN, T., RANKI, A. & HAYRY, P. (1979) Morphological and functional characterization of isolated effector cells responsible for human Natural Killer activity to fetal fibroblasts and cultured cell line targets. *Immunol. Rev.*, **44**, 71.

SHAALA, A. Y., DHALIWAL, H. S., BISHOP, S. & LING, N. R. (1979) Ingestion of dyed-opsonised yeasts as a simple way of detecting phagocytes in lymphocyte preparations: Cytophilic binding of immunoglobulin by ingesting cells. *J. Immunol. Methods*, **27**, 175.

SPIERS, F. W. (1968) $^{90}$Sr in autoradiographic studies in bone. In *Radioisotopes in the Human Body: Physical and Biological Aspects.* London: Academic Press. p. 217.

TALMADGE, J. E., MEYERS, K. M., PRIEUR, D. J. & STARKEY, J. R. (1980) Role of NK cells in tumour growth and metastasis in beige mice. *Nature*, **284**, 622.

WARNER, N. L., WOODRUFF, M. F. A. & BURTON, R. C. (1977) Inhibition of the growth of lymphoid tumours in syngeneic athymic (nude) mice. *Int. J. Cancer*, **20**, 146.